# 

**Published:** 1890-11

**Authors:** 


### SPECIAL.

We send out with the current number, to such of our subscribers as are in arrears, a statement of their indebtedness, and request that they, one and all, forward the amount to this office. We feel sure that it is owing to carelessness on their part and neglect on ours that these small accounts have not been attended to before this time.

We would call special attention to our premium list, which has been selected with especial regard to the value and usefulness of the several works mentioned.

We publish no clubbing list, but any person desiring to subscribe for our journal in connection with any other publication extant, will receive them at the fullest discount attainable.

### LITERARY.

THE MONEST is the title of a new quarterly magazine, issued by the Open Court Publishing Co., of Chicago. The high literary and scientific character of its regular contributors furnish a sufficient guarantee of the prospective value of this new candidate for the favor of readers and thinkers above the commonplace, irrespective of the manifest metaphysical tendency of some of the essayists in dealing with themes which, from their nature, lead into the region bordering upon speculation and conjecture.

In point of materials and mechanical execution this quarterly is all that even Chicago could reasonably ask of it.

SYNOPSIS OF A COURSE IN MICROSCOPY FOR PHARMACISTS.—By Dr. H. M. Whelpley, F. R. M. S., intended as a guide for a course of home study.

GOOD HOUSEKEEPING.—This excellent family paper, after next January, is to be issued monthly, instead of fortnightly, as heretofore. It is about to publish "The Romance of a Thanksgiving Turkey," but *our* readers are better satisfied with the *reality!*

THE CENTURY MAGAZINE advertises the life of Abraham Lincoln, by Nicolay and Hay, and the autobiography of Joseph Jefferson, which so recently enriched its serial columns, in book form ; two excellent publications.

YOUTH'S COMPANION.—OUR LITTLE MEN AND WOMEN, AND BABY-LAND.—are preparing to dazzle their youthful patrons with gorgeous holiday numbers.

